# Validation of the Italian Version of the Caregiver Abuse Screen among Family Caregivers of Older People with Alzheimer's Disease

**DOI:** 10.1155/2017/3458372

**Published:** 2017-02-07

**Authors:** Maria Gabriella Melchiorre, Mirko Di Rosa, Francesco Barbabella, Norma Barbini, Fabrizia Lattanzio, Carlos Chiatti

**Affiliations:** ^1^Centre for Socioeconomic Research on Ageing, National Institute of Health and Science on Ageing (INRCA), 60124 Ancona, Italy; ^2^Scientific Direction, National Institute of Health and Science on Ageing (INRCA), 60124 Ancona, Italy; ^3^Department of Health and Caring Sciences, Linnaeus University, 39182 Kalmar, Sweden; ^4^Epidemiological Observatory, National Institute of Health and Science on Ageing (INRCA), 60124 Ancona, Italy

## Abstract

*Introduction*. Elder abuse is often a hidden phenomenon and, in many cases, screening practices are difficult to implement among older people with dementia. The Caregiver Abuse Screen (CASE) is a useful tool which is administered to family caregivers for detecting their potential abusive behavior.* Objectives*. To validate the Italian version of the CASE tool in the context of family caregiving of older people with Alzheimer's disease (AD) and to identify risk factors for elder abuse in Italy.* Methods*. The CASE test was administered to 438 caregivers, recruited in the Up-Tech study. Validity and reliability were evaluated using Spearman's correlation coefficients, principal-component analysis, and Cronbach's alphas. The association between the CASE and other variables potentially associated with elder abuse was also analyzed.* Results*. The factor analysis suggested the presence of a single factor, with a strong internal consistency (Cronbach's alpha = 0.86). CASE score was strongly correlated with well-known risk factors of abuse. At multivariate level, main factors associated with CASE total score were caregiver burden and AD-related behavioral disturbances.* Conclusions*. The Italian version of the CASE is a reliable and consistent screening tool for tackling the risk of being or becoming perpetrators of abuse by family caregivers of people with AD.

## 1. Introduction

Elder abuse has been defined as “*a single or repeated act, or lack of appropriate action, occurring within any relationship where there is an expectation of trust, which causes harm or distress to an older person*” [1]. It can be sexual, physical, psychological, and financial and could also assume the form of neglect [2, 3]. Elder abuse is a growing concern among practitioners and policy makers in the long-term care sector in many countries. Although it is still difficult to understand its true prevalence, with the increasing ageing population, the potential number of victims is likely to increase [4, 5].

The main risk factors for elder abuse are universal across countries (e.g., United States, Canada, and European Union) and are related to specific characteristics of the victims. These include cognitive impairment, dementia, poor mental health, depressive symptoms, problematic-aggressive behavior, physical disabilities, financial difficulties, social isolation, and lack of social support [4, 6–12]. Cultural acceptance or tolerance of ageism and violence can also be a driver for elder mistreatment in some areas [4]. Other potential risk factors are known to be sensitive to change across different national contexts, for instance, victim's age and gender [12]. In the United States, younger age is associated with higher risk of elder abuse [13, 14], whereas studies in Europe showed that older age groups are at greater risk [10, 15]. Studies from Ireland [10], Israel [16], and United Kingdom [17] reported that the larger number of victims is women. Different results were found instead in other countries, where men are more likely to experience abuse in later life, especially financial and emotional one, like in Korea [18], and in other European countries [19]. In the United States [13] gender was not identified as either a risk or a protective factor.

Research findings on risk factors of perpetrators of elder abuse are still limited [12]. We know that, across different countries, perpetrators are most likely to be adult children or spouses, male, to have history of substance abuse, to have poor mental or physical health, to be socially isolated and unemployed, and to experience high levels of care burden [3, 7–9]. Caregiver risk factors specifically associated with elder abuse of people with dementia are poor psychological health, mental health problems (e.g., depression and anxiety), and alcohol abuse [20]. Likewise, low self-esteem has been reported as a predictive factor for violent behaviors [21].

In Europe, the prevalence of psychological and verbal abuse ranges from 0.3% in Spain [22] to 3.2% in the Netherlands [23], but it is even higher in the case of people with dementia, ranging from 34% to 62% [24, 25]. Older adults with Alzheimer's disease (AD) were found to be 4.8 times more likely to report elder abuse by their caregivers when compared to those without dementia [26]. However, the intrinsic mechanisms underlying this issue have not yet received much attention [27, 28]. This is even more worrying considering that around 47 million people worldwide are living with dementia, and this percentage is likely to almost double every 20 years (about 75 million in 2030 and 132 million in 2050) [29].

The prevalence of elder abuse in Italy is difficult to estimate, but integrating several sources a rate around 10–14% has been proposed [30], which is the lowest among seven European countries according to the results of the ABUEL study [31]. The risk of psychological abuse and financial mistreatment are higher in the domestic settings, with lower rates of physical abuse and neglect. A comprehensive, national survey of violence against older people [32] suggested that 9% of older respondents were exposed to psychological abuse and 3% to fraud and theft, while only 1% experienced either physical abuse or neglect. The ABUEL study confirmed these results [33, 34] and highlighted psychological and financial abuse as the principal forms (about 11% and 3%, resp.), whereas very few cases of physical (1%) and sexual (0.5%) abuse and neglect (0.7% on the whole) were detected. Older Italian women are at higher risk of physical, sexual, and psychological abuse (perpetrated frequently by a male, e.g., a former partner) than older men. The ABUEL study also showed a higher prevalence of Italian male victims who reported mainly psychological and financial episodes [33]. “Irregular situations” within institutional care are also frequently reported by the media [30]. These are usually related to the use of expired medications, poor hygienic conditions and malnutrition. Likewise, abusive situation in form of fraud and thefts are often reported in the Italian media, however, the true relevance of the phenomenon is likely to be underestimated as the financial exploitations occurring within the household, could be often hidden as older people consider almost “natural” the idea to support economically their children or close relatives, and thus they do not perceive this behavior as abusive [30]. In case the perpetrators are family members, also emotional reasons could prevent older victims from reporting episodes of mistreatment.

On the whole, in Italy elder abuse still represents a “social taboo” hard to tackle, as it tends to remain hidden within familial boundaries. Large part of the abusive episodes, which are often associated with the caregiving burden, often remain unrecognized. This is due to a lack of awareness about what elder abuse concretely is and it is also caused by the systematic under-reporting of abuse episodes by older people themselves, especially when the perpetrator is a family member on whom they are depending for care and support [30]. Indeed, the traditional engagement of family caregivers, the high costs of care in nursing home, and the lack of adequate welfare policies supporting the victims of elder abuse [35] have led to the widespread situation where many older people still live with their adult children, who take care of them often in stressful conditions (trying to conciliate family, private life, and caregiving activities). In such a context episodes of violence can easily occur. In addition, the lack of qualified care staff trained in recognizing elder abuse affects the reporting too [30], and the lack of an appropriate legal framework at national level represents a major barrier for addressing this phenomenon in the country [35, 36].

The lack of comparable data on elder abuse is a further issue in Italy, and it is related to the heterogeneity of the studies performed in the field: available studies have indeed used different definitions and methodological approaches (e.g., sampling methods, recruitment procedures, study designs, and measures). Most of the studies did not use validated tools but relied on quantitative or qualitative ad hoc questions and focus groups [30]. In few cases, the Minimum Data Set for Home Care interview (MDS-HC), including measures to detect potential abuse (e.g., older person is fearful and shows poor hygiene, neglect, unexplained injuries, and signs of physical restraint), was used (e.g., [37, 38]). Most studies excluded people with dementia, as their impaired mental capacity prevented them from answering the questions in the instrument used for the survey. This is also the case of the ABUEL study [33], which assessed violence using, for instance, a 52-item instrument derived from a previous study in the UK [39], and systematically excluded people with dementia.

Available literature proposes many instruments to detect cases of elder abuse [40], focusing on different dimensions. Elder abuse screening instruments can be generally organized into three groups: (1) screening instruments based on direct questioning to the potential victim, (2) instruments inspecting for signs of abuse, and (3) instruments evaluating the overall risk for abuse [41–43]. Instruments have also been categorized as qualitative and/or quantitative [8, 44–46], as being combined with assessment protocols and guidelines [9] or being a stand-alone instrument, simply based on a list of items [47]. Additional tools used in this area, which are based on different approaches, are the Brief Abuse Screen for the Elderly (BASE) [48]—with five questions screening older people who are caregivers or care recipients and detecting potential abuse in both ways—and the Conflict Tactic Scale (CTS) in the original [49] or adapted version [50]—used for identifying and measuring family violence and various forms of abuse.

When it comes to the dementia field, screening methods could be even more challenging. It is known that direct questioning approach is difficult to use, while screening methods collecting information from different sources could be more effective [41–43, 51, 52]. In this situation, the Caregiver Abuse Screen (CASE) tool [53] seems one of the best instruments available. It identifies family caregivers possibly guilty or at risk of becoming perpetrators of physical, psychological abuse or neglect, by asking questions directly to caregivers themselves. This tool can only indicate a possible elder abuse behavior, without diagnosing it [43], and it was originally developed within the Project CARE in a local community-based health and social service agency (CLSC NDG/Montreal West) in Canada, in order to identify suspected or potential abusive caregivers and related victims and to provide preliminary intervention.

Despite the fact that several translated versions exist in languages other than English (e.g., Spanish and Brazilian Portuguese), the CASE has never been translated and validated in Italy.

The main aim of this paper is to validate the Italian version of the CASE tool in the context of dementia caregiving. In addition, this work explores factors associated with elder abuse risk in Italy.

## 2. Materials and Methods

### 2.1. Data

The study draws on data from the baseline assessment of the Up-Tech study, a 12-month randomized controlled trial (RCT) aimed at evaluating innovative care services for older patients with moderate AD and their family caregivers living in the community. The study protocol is available elsewhere [54], as well as its first results [55–57]. The inclusion criteria for patients were (1) being 65 years old or more, having moderate AD—evaluated by a Mini-Mental State Examination (MMSE) score between 10 and 20 [58], (2) living at home, and (3) being cared for by at least one family caregiver. The primary caregiver was defined as that relative providing support to the patient in activities of daily living (ADLs) and/or instrumental activities of daily living (IADLs) for at least 1 hour per day in the last 6 months on average.

Dyads of older people with AD and their primary family caregivers were recruited by using lists of patients available at each Alzheimer Evaluation Unit in five health districts of the Marche Region (Pesaro, Ancona, Macerata, Fermo, and San Benedetto del Tronto). An invitation letter was sent to 640 dyads, and 438 of them were recruited [59], with a response rate of 68%, corresponding to the number of complete interviews, divided by the total number of interviews plus refusals, break-offs, noncontacts, and all other cases of unknown eligibility [60].

### 2.2. Measures

Up-Tech questionnaire was based on the interResident Assessment Instrument Contact Assessment (interRAI CA) [61] and included several measures on demographic and socioeconomic aspects (ad hoc questions), as well as physical and psychological health issues (mainly validated and standardized measures). It was administered by trained research nurses to patients and family caregivers, with the latter ones acting as proxy if the patient could not answer by himself/herself.

Patients' functional status was assessed using the ADL Hierarchy Scale [62] and the IADL Scale [63]. Cognitive impairment was assessed using the MMSE, while we used a yes/no question to the family caregiver to evaluate if the older person with AD suffered from behavioral disturbances.

Caregivers' conditions have been extensively assessed. Mental health was measured using the Short Form Health Survey (SF-12), which distinguishes a Mental Component Score (MCS-12) and a Physical Component Score (PCS-12) [64, 65]. Caregivers' burden was assessed via the Caregiver Burden Inventory (CBI) [66, 67], while for depressive and anxiety symptoms we used the Hospital Anxiety and Depression Scale (HADS) [68] and its two related subscales. Social support was measured with the Multidimensional Scale of Perceived Social Support (MSPSS) [69], which is structured in three subscales concerning support from family, friends, and others.

We used the CASE instrument [53] to assess the risk of elder abuse potentially perpetrated by the caregiver. It requires only one-two minutes to be completed, and includes 8 items in form of binary (yes/no) questions with a score ranging from 0 to 8. A value higher than 4 indicates high risk of abuse. Each item (described in Table 3) explores possible cases of physical, psychological abuse or neglect, without asking the caregivers for specific abusive behaviors. Previous validation studies [52, 53] identified two components representing

- neglect (items 5 and 7): that is, the caregiver feels tired/exhausted and thus he/she cannot do what is necessary/to be done to meet the needs of the older person;
- interpersonal (physical/psychological) abuse (items 1, 2, 3, 4, 6, and 8): that is, the caregiver feels forced to act out of character or to be rough with the older person, or he/she has sometimes trouble in controlling his/her temper/aggression towards the older person. In particular, the wording of this last question is consistent with the* Control Theory*; that is, “the perpetrator's belief in external locus of control has predicted the potential abuse” ([53]: p. 48).

On the whole, the wording of the eight items of CASE has a nonblaming approach [70]; that is, they are consistent with the* Neutralization Theory* which explains mistreatment as involving justifications by the potential abuser (e.g., the caregiver feels too tired and at the same time feels that reducing the care to the older person may be acceptable) [53].

The original CASE tool was translated into Italian using the following steps. In first place, the forward translation of the CASE, from English to Italian, was carried out by two independent researchers with a very good knowledge of both languages. The two translations were compared and the most acceptable option was agreed. Then, a back-translation from Italian into English by a third translator (English mother tongue and fluent in Italian) confirmed the consistency of the translated version.

### 2.3. Data Analysis

Descriptive statistics for the sample were calculated, for the caregiver and the older person with AD, in terms of percentage distribution for categorical variables and in terms of means and standard deviations (SD) for continuous variables. We explored the bivariate associations between the CASE total score and subtypes of abuse identified by previous literature [52, 53] and the sociodemographic and health-related characteristics of the sample. For continuous variables, Pearson's correlation coefficient (*r*) was calculated. For categorical variables, CASE mean scores were first computed for each category; then, *t*-test and Kruskall-Wallis test were performed, respectively, for binary variables (gender and behavioral disturbances) and for the other categorical variables (education and caregiver-older person relationship). The statistical significance for all the bivariate analyses was set at *p* < 0.05.

In order to validate the Italian version of the CASE, we first computed Spearman's rank correlation coefficients with Bonferroni-adjusted significance level for each CASE item, in order to investigate their internal correlations. We then performed a principal-component factorial analysis with varimax rotation, in order to evaluate the factor validity of the CASE tool and to verify the existence of one or more underlying factors. According to Kaiser Criterion, only those factors with eigenvalues equal or higher than 1 were retained. Cronbach's alphas were also calculated to test the reliability and internal consistency of the scales (full scale and subscales as emerging by the analysis or already suggested by the literature) [52, 53]. Finally, we performed a multivariate linear regression analysis in order to assess the construct validity of the instrument and to identify the main risks of elder abuse in the Italian sample. In order to know whether data satisfied parametric assumptions, we used the Breusch-Pagan/Cook-Weisberg test for heteroskedasticity, which accepted the null hypothesis (*p* = 0.6087), therefore the constant variance assumption was accepted too. Likewise, the *p* value of a Shapiro-Wilk test for normal data was greater than .05; therefore we did not reject that residuals were normally distributed. Moreover, in order to detect the collinearity of the regressors with the constant, variance inflation factors (VIFs) were calculated confirming that no collinearity issue can be raised. The total score of the CASE was calculated as the sum of positive response in each component, thus ranging from 0 for “no risk” to 8 for “high risk” of abuse. CASE total score was assumed as dependent variable, and all variables resulting in associating it with statistical significance in the previous bivariate analysis were assumed as independent variables in the model, together with the control variables of age and gender (for both the caregiver and the older person). Multivariate regression model was elaborated also with regard to two subtypes of abuse, which were identified within the CASE test by previous literature [52, 53], in order to verify how independent variables influence each subscale. The scores of CASE subtypes (as sum of positive responses) were the following: 0–6 for interpersonal/physical-psychological abuse (CASE items: 1, 2, 3, 4, 6, and 8), and 0–2 for neglect (CASE items: 5 and 7). The statistical significance of the coefficients was assessed by a *t*-test, and it was set at *p* < 0.05. The validity of the model was verified with the F-test of joint zero coefficients and their explanatory power by the R^2^. Analyses were performed using STATA, version 11.2 (Stata Corp., College Station, TX).

### 2.4. Ethics

The study was conducted in accordance with the Declaration of Helsinki (1964, as amended to 2013) and was based on voluntary participation. Informed consent was asked from both older people (if still in their capacity) and family caregivers in the sample. The study protocol was submitted to and approved by the competent Marche Regional Ethical Committee.

## 3. Results

### 3.1. Descriptive Statistics

The majority of older people with AD enrolled were composed of women (71.5%), with a high mean age (81.5 years) (Table 1). Cognitive impairment was moderate (MMSE 16.2 ± 3.3), with around a quarter of patients suffering from behavioral problems. Difficulties in IADLs were already high in the sample (35.2 ± 13.4), whereas ADL resulted in being quite intact (1.5 ± 1.6). Family caregivers were mostly women (66.2%) over 60 years old. Over half of them were child or child-in-law of the patient. Caregivers had prevalently a low educational levels (30.1% had no formal title/basic level education, and 24.9% completed only the primary school) and showed a moderate level of social support (MSPSS 62.1 ± 13.8), especially from family and others. CASE total score was relatively high and equal to 3.7 (±2.8).

### 3.2. Bivariate Analysis

Preliminary risk factor analysis was elaborated with the bivariate analysis between the screening tool and the sociodemographic and health-related characteristics of the caregiver-older person dyad. The risk of abuse (CASE total) perpetrated by the family caregiver to the older person with AD (Table 1) was positively and significantly correlated with the following variables: anxiety (*r* = 0.38) and depression (*r* = 0.35) of the caregiver (HADS subscales); caregiver burden (*r* = 0.55) (CBI); presence of behavioral disturbances (mean = 5.22); and IADL limitations (*r* = 0.20) of the cared-for person. Conversely, CASE was inversely correlated with social support perceived by the caregiver (MSPSS), in general (*r* = −0.18), but especially that coming from family or friends (*r* = −0.16), and caregiver's overall physical (*r* = −0.17) and mental health status (*r* = −0.38) (PCS-12 and MCS-12, resp.). CASE total score was also positively and significantly correlated with the female gender of the caregiver, suggesting that women, who take care of their older family members, could be at higher risk of being/becoming perpetrators of abuse. On the whole, the associations found with the CASE total score were consistent also with those concerning both subtypes of abuse (interpersonal abuse and neglect), thus showing a difficulty in making a clear distinction between them.

### 3.3. Correlations between CASE Items

Analysis of Spearman's rank correlation coefficients between CASE items (Table 2) revealed that, except for two pairs of correlations (between item 5 and item 8, and between item 4 and item 7, resp.), all items were correlated with moderate or high coefficients (ranging from 0.34 to 0.65). Furthermore, all coefficients were statistically significant (*p* < 0.001).

### 3.4. Factor Validity

All caregivers completed the CASE instrument (Table 3). The results indicated the prevalence of potentially abusive behaviors in the sample: 60.7% of respondents reported the feeling of being forced to be rough with the older person, while in 54.6% of cases the caregivers reported to yell at their relatives with AD. The results of the factor analysis performed on these items suggested the presence of a single underlying factor, with Factor 1 having an eigenvalue greater than 1 (4.03, *p* < 0.001). Cronbach's alpha of the eight components of the CASE (full scale) was 0.86, thus showing high reliability. Since previous validation studies of other CASE versions identified two principal components, representing neglecting behaviors (items 5 and 7) and physical/psychological abuse (items 1, 2, 3, 4, 6, and 8), we calculated the Cronbach's alpha coefficients for these subscales, which resulted equal to 0.53 and 0.84, respectively. Furthermore, 50% of communalities were >0.50 (ranging from 0.43 to 0.63).

### 3.5. Construct Validity

Construct validity of the Italian version of the CASE was assessed by verifying the relations of CASE total score and subtypes of abuse with other variables associated with risk of elder abuse. In addition to the bivariate analysis already described above, a multivariate linear regression analysis was performed for identifying factors associated with abuse risk (Table 4). In the regression model, we excluded the following variables: MMSE and ADL because they were not statistically significant in the bivariate analyses (Table 1), and moreover due to collinearity issues, respectively, with behavioral disturbances (Spearman's rho = −0.0984, *p* = 0.0396) and IADL (rho = 07525, *p* = 0.0000). The total *R*-squared of the model was equal to 0.34. With regard to CASE total score, it showed that only CBI (*β* = 0.08) and behavioral disturbances (*β* = 1.47) increase the risk of abuse in a statistically significant way, whereas IADL limitations seem to reduce the risk of abuse (*β* = −0.03; *p* = 0.008). IADL, behavioral disturbances, and CBI emerged as statistically significant also in determining the value of each CASE subscale, just like for the total scale. In addition, MSPSS and HADS anxiety emerged as statistically significant for increasing the risk of the neglect subscale.

## 4. Discussion

The original study [53] and the Spanish version [52] validated the CASE showing the existence of two principal components: one for neglect (items 5 and 7) and one for interpersonal (physical/psychological) abuse (items 1, 2, 3, 4, 6, and 8). Cronbach's alpha coefficient for the full scale, respectively, of 0.75 and 0.84 was found. Conversely, the validation of the Portuguese version in Brazil [71] proposed a one-factor analysis, instead of the abovementioned two-factor explanation, with a Cronbach's alpha coefficient of 0.85. In line with this latter evidence, our one-factor structure showed high reliability and internal consistency (Cronbach's alpha = 0.86), in addition to good Spearman's rank correlation coefficients of the CASE items (all statistically significant ranging from 0.34 to 0.65). Our study seems to confirm that, at least in the Italian context, the neglect component cannot be clearly distinguished from the interpersonal (physical/psychological) abuse factor.

Some cautious interpretations can be proposed to explain why in Italy a single factor structure for the CASE has been found, in line with the validation of the Portuguese version in Brazil. We can hypothesize that cultural issues influence the responses of interviewees: in Italy the phenomenon is still hard to be detected and occurs mainly in the form of psychological and financial abuse, and less as neglect and/or physical abuse. Thus, we can suppose that neglect here cannot emerge as second factor in the CASE structure, as a consequence of the low awareness of caregivers. In a previous study, we actually found only 0.7% of neglect episodes in the country [34]. In order to better distinguish neglect from other categories of abuse, the inclusion of additional items in the CASE tool addressing this issue has been proposed [52], as well as an adjustment of the wording of the items themselves [71].

The need to address more thoroughly neglect is indeed of high relevance. A recent scoping review on elder abuse from an international perspective [12] pointed out that a 1-year neglect prevalence ranged from 0.2% to 5.5% (in Europe, 0.5%). Interestingly, the same review showed that most studies used unstandardized instruments for screening neglect and different definitions of neglect itself (e.g., as one or more negligence events within a given time period or as a minimum of 10 events in the past year). These circumstances stress the importance of instruments for assessing elder abuse, and for detecting neglect in particular. Moreover, given that in Italy we observe mainly financial exploitation (besides psychological abuse) which is not currently covered by the CASE, it seems necessary to work on new items to add in order to address such relevant issue. Two items involving financial abuse were included in the original version of this screening instrument, but they were then dropped because their response rates were similar when comparing potential abusers and nonabusers [53]. Also in this case, an adjustment in the wording could be useful. Finally, given the different results obtained in different countries in validating the CASE (i.e., one or two of principal components), maybe issues concerning different cultural-linguistic backgrounds should be considered more deeply [71].

Nevertheless, the construct validity of the Italian version of CASE tool is supported by the bivariate relations found between the CASE total score (and subtypes of abuse, that is, interpersonal abuse and neglect) and those variables that literature suggested to be associated with elder abuse. We found positive significant relations between risk of elder abuse and caregiver characteristics, such as female gender, anxiety, depression and burden, but also with AD patient's characteristics, such as the presence of behavioral disturbances or IADL limitations. Our results also showed negative significant associations between higher risk of abuse and less perceived social support of the caregiver, in addition to the caregiver's overall physical and mental health status. When multiple explanatory factors of risk of abuse were evaluated together by means of multiple linear regression, and with regard to both CASE total score and subtypes of abuse, only the burden of the caregiver and behavioral disturbances of the older person significantly increased the risk of abuse. IADL limitations of the older person seemed conversely to reduce the risk of abuse when all dimensions are all included in the regression analysis. Interestingly, in the multivariate analysis the perceived social support and anxiety of the caregiver emerged as statistically significant for increasing the risk of neglect.

With regard to dimensions which are statistically significant only in the bivariate analyses and concerning CASE total score, depression and anxiety as caregiver risk factors for elder mistreatment are well documented in the literature, especially among older people with dementia [72–76]. Our findings, showing negative significant correlations between less perceived social support of the caregiver and more risk of abuse, also are confirmed by other studies [77–80]. Finally, the negative correlation between worse caregiver health status and higher risk of abuse is consistent with existing evidence [8, 70, 81–84]. The fact that the CASE total score was positively and significantly associated with the female gender of the caregiver is also supported by literature, showing that, although generally we have mainly potential male perpetrators across countries [3, 9], when exploring informal caregiving, women show a higher risk of committing elder mistreatment than men [6, 85], and they are indeed often the primary caregivers of older people [86]. Women further experience in general greater burden and work restrictions than men due to caring responsibilities [87].

With regard to variables which remain statistically significant in the regression analysis, concerning both CASE total score and subtypes of abuse, the caregiver burden is indeed often indicated also in the literature as a relevant risk of mistreatment [7, 8, 46, 47], particularly in dementia caregiving [88–90], and cognitive impairment of the older person was found to be related to the risk of abuse [77, 91], especially in presence of provocative and aggressive behaviors of older people with dementia [6, 24, 74, 75, 92]. We should also consider that dementia represents itself as a relevant risk factor for elder abuse, and family caregivers perceive the related aggressive acts as crucial challenges to tackle [20, 93, 94].

The results regarding burden of the caregiver and behavioral disturbances of the older person, which showed a significant increase in the risk of abuse, are also supported by other studies highlighting how behavioral disturbances in patients with dementia are the main risk for institutionalization and acute hospitalization [95], leading to great distress and burden for family caregivers [96–99]. High level of burden experienced by caregivers might thus result in higher risk of elder abuse, especially among older persons with behavioral disorders [100]. In particular, associations between agitated behaviors of older people, caregivers' burden, and verbal and physical abuse by family caregivers were found [101]. The likelihood of abuse can thus be a reaction of the carers, especially when stressed and depressed, to problematic assault and aggressive behaviors by the older person [75, 97, 102]. However, it has also to be considered the probability that behavioral disturbances could be the response of the older victims to physical or sexual abuse and neglect. Therefore, it is difficult to identify causal pathways in this context and people with dementia may either act and/or receive physical abuse. Thus, a sort of mutual violent behaviors from perpetrator to victim and vice versa seems conceivable in these contexts. Victims with dementia may act (provoked or not) and receive physical abuse [26]. It is anyway to stress that although majority of caregivers refer to be overburdened, this does not imply that all are or could become potential abusers of their older relatives [74], given that burden is a complicated and multidimensional aspect involving physical, psychological, economic, and social issues [103].

IADL limitations of the older person seem conversely to reduce the risk of abuse when all dimensions are included in the regression analyses. In this respect literature shows mixed evidence concerning the association between level of functional impairment in older people with dementia and elder abuse, with some studies confirming the relation [88, 104] and others not supporting it [21, 24, 73]. In particular, two studies, which performed multivariate analysis to explore risk factors for elder abuse of demented older people, found that caregivers were less likely to abuse those with greater functional impairment [105, 106]. In addition, Johannesen and Logiudice [84] highlighted that functional impairment of older people with dementia does not represent a risk factor for mistreatment, when compared to studies involving general population. In the case of dementia, cognitive impairment and related aggressive behaviors are more burdening than physical impairment, and thus further disability condition of the older on the whole does not add to the already high risk of abuse. On the whole, many studies report indeed that caregiving for people with dementia is more stressful than caring for people with functional limitations [107–109].

Furthermore, no association was found between the risk of elder abuse and less perceived social support in the regression analysis, with regard to CASE total score, whereas conversely this emerged from the bivariate analysis. This result is of difficult interpretation, given that most previous literature supports this relation strongly and consistently, as mentioned above [77, 78]. Some studies highlighted that abusive caregivers suffer from social isolation and low social support to assist them with their caregiving tasks, which are often leading to increased burden levels due to high levels of needs among victims [4]. In particular, Kilburn Jr. [80] found that instrumental and emotional support from network contacts, for sharing caregiving experience, reduced the probability of violent attitude of the caregiver towards the care-recipient. Moreover, a further study showed that only perceived social support, but not instrumental, was linked with increased risk of elder abuse [110]. Other studies did not show significant associations between elder abuse and levels of social support or isolation [21, 52, 94]. This inconsistence of our results might be partly explained by the sampling strategy of the Up-Tech study. Since we recruited patients from the Alzheimer Evaluation Units (and thus they were supported from services) and the participation to the study was voluntary (potential selection bias), family caregivers in our study might be characterized by a higher level of perceived social support that has not been detected by the MSPSS test we used in this respect (support from family, friends, and significant others), and thus this context might be the reason why no association with risk of elder abuse was found in a multivariate exploration.

Finally, perceived social support and anxiety of the caregiver were statistically significant in the regression analysis with regard to neglect subtype.

Anxiety is indeed a crucial risk factor for elder abuse, including neglect [72–76]. In particular, Reay and Browne [111] investigated the differences in risk factor characteristics between caregivers who physically abused their older relatives and caregivers who neglected them. They found indeed higher depression scores in the first group, whereas higher anxiety levels emerged in the second one. Wiglesworth and colleagues [75] also found higher significant mean value of anxiety among caregivers neglecting older adults (without physical abuse), when compared to other types of mistreatment (even though the post hoc test for this relation was not significant). Probably, when the caregiver of the older person is anxious and distressed [107, 112, 113], he/she is also more prone to neglect the cared-for person and to address inappropriately care needs [114]. However, also a reverse pattern is possible, that is, neglect, intended as failure to meet the needs of the older person [12], can generate anxiety in the caregiver, due to incapability to take care and to provide adequate assistance. This could be more evident in case of a dementia caregiving, where the older persons could be unable to communicate their own needs and consequently the caregiver could be unable, for instance, to understand and address nutritional and pain issues [115, 116].

Also the fact that a social support perceived by the caregiver seems to increase the risk of neglect could be explained by the particular nature of this type of mistreatment. Neglect is a “nonaction”; that is, it concerns ignoring the older people in need of care. A caregiver with a supportive social network could trust in the help from other persons or services and thus he/she could feel less the burden to be the only responsible for care. This circumstance could in turn lead to neglect because there will probably someone else, apart from the (primary) caregiver, who will provide the necessary care. Conversely, physical and psychological abuse are violent actions and a lower social support could have no significant effect or even increase their risk, as emerged, respectively, in our multivariate and bivariate analyses. Anyway, other research findings [117] showed that, with regard to mistreatment in general, family caregivers with high social support could abuse their older disabled relatives like caregivers without any support network.

On the whole, with regard to the particular relation between social support and neglect, we found ambivalent results in our study (e.g., negative association in the bivariate analysis, and positive association in the regression analysis). This further suggests that neglect is a subtype of abuse that needs separate and more accurate exploration, by adjusting wording and integrating further items in the CASE tool, as already highlighted. Currently, in the CASE test, neglect is assessed only by means of two items and this seems insufficient in order to evaluate more deeply this peculiar form of mistreatment and the related risk. In fact, as Sev'er highlights [118], neglect of older people is the most common form of mistreatment, but it is not easy to prove its existence.

### 4.1. Limitations

This study had some limitations to take into account. First, this is only a preliminary validation of the Italian version of the CASE with a quite specific sample (family caregivers of older people with moderate AD). Second, the CASE tool does not assess financial abuse, indeed leaving out a relevant dimension of abuse. Third, CASE is easy to be quickly administered due to its brevity [53], but brief screening tools usually cannot catch the complexity of individual behaviors and relationships [41]. Fourth, as already mentioned, patients and caregivers of the Up-Tech study were recruited from the Alzheimer Evaluation Units. This implies that our sample was composed by people already followed by formal care services, factor which might mitigate the risk of potential abusive situations. Finally, although CASE is a useful instrument to explore potential elder abuse by carers when victims cannot answer, as it is the case of older people with dementia, the “missing voices” of older persons themselves represent anyway a crucial gap in this kind of research.

### 4.2. Conclusions

Our study validated the Italian version of the CASE in the context of family caregivers of people with moderate AD and confirmed that it is a reliable and consistent screening tool for tackling the risk of being or becoming perpetrators of elder abuse by family caregivers. In particular, caregiver burden and AD-related behavioral disturbances of the older person were found significantly increasing the risk of abuse. The validation of this tool in Italy is extremely important, given the CASE comprehensive nature of including relations with dimensions of the older person, the potential perpetrator, and concerning the context of the situation, as it has already been recently provided, for instance, in Spain [52] and in Brazil [71]. Our study also highlighted that, in Italy, the neglect component does not emerge clearly distinguishable from the interpersonal (physical and psychological) abuse factor. In this country, indeed, elder abuse still remains a “social taboo” hard to be detected and it is hidden both within familial boundaries and institutions. Older adults usually do not report episodes of violence, especially when they depend on relatives for care and support. Moreover, the phenomenon is mainly perceived and reported as psychological and financial abuse and less as physical abuse and neglect.

In terms of practical and policy implications, our study highlights that having empirically tested, evidence-based, and valid screening instruments for assessing potential elder abuse represents a crucial preliminary step in order to obtain estimates of the extent and risks of elder abuse, which are in turn essential for designing appropriate interventions and policy planning [119, 120]. The CASE tool can anyway be of great help only as initial exploration of caregivers' behavior and attitude towards elder abuse, and should suggest eventual further examination and even the integration of complementary screening tools for a deepest evaluation of the suspicion of violence, when positive answers emerge from the administration [121]. In this respect, professionals (e.g., doctors, nurses, and social workers) should be put in the condition of receiving adequate training on elder abuse by family members and how to detect it, recognizing evident signs and administrating relevant screening tools.

The CASE could also be used to prevent real abuse and for early intervention when a potential abusive situation is suspected. Early detection of elder abuse is thus important, but the efficacy of routine screening will depend on effective and proactive approach to interventions, which should lead to reporting by the potential victims and to cooperation between professionals for managing the cases of mistreatment eventually detected. In particular, interventions for supporting potential abusive caregivers and relieving them from burden (e.g., housekeeping, meal preparation, respite care, support groups, and day care) seem a “promising approach” to elder abuse prevention [12]. Caregivers themselves of older people with dementia have referred that home and respite care services are important supports that could be of help for preventing potential abuse [122]. Furthermore, also proactive interventions aiming at education and training of population on elder abuse issues should be considered. In Italy, there is a lack of a national policy on elder abuse, and it is therefore urgent to identify and implement measures which might be effective to prevent and report elder abuse and in particular to support informal caregivers in assisting their dependent older relatives [30].

Moreover, interventions need to be integrated with ongoing research, evaluation, and capacity building [70]. With specific regard to CASE, also future research is needed first of all in order to provide more psychometric evaluation of this test, in Italy and in other countries, and also to examine whether its item scores are associated with potential risk of abuse, in the light of some multivariate associations with expected external dimensions which are not supported by our study (e.g., social support and CASE total) or emerge only with regard to a subtype of abuse (e.g., social support and neglect). Further validation of this tool in different cultural-linguistic contexts could also address “cross-cultural measurement equivalence” ([71]: p. 881). It should indeed be a priority to understand better the cultural issues related to elder abuse in different populations [123]. The inclusion of further items addressing potential risk of neglect and financial abuse could be of help for better detecting of more forms of mistreatment. The necessity of this inclusion is also indirectly supported by studies which put in evidence a relation between economic situation, exploitation and neglect of the older person. In particular, older people without financial strain could be exposed to financial abuse [124], whereas vulnerable older people with a poor economic situation could be victims of neglect [125].

To understand circumstances and mechanisms increasing the likelihood of elder abuse by means of CASE test is thus very important, also because some risk factors can represent useful indicators for primary prevention and intervention [126]. In this respect, to know the potential perpetrators' characteristics and relations with victims seems absolutely relevant [127].

## Figures and Tables

**Table 1:** Sociodemographic, health, and psychological characteristics of the sampled dyads (*N* = 438) by CASE total and subtypes of abuse.

	*N*	%/mean (SD)	CASE total *r*/mean (SD)	*p* value	CASE interpers. *r*/mean (SD)	*p* value	CASE neglect *r*/mean (SD)	*p* value

*AD patients' characteristics*								
Gender								
Male	125	28.5	3.89 (2.8)	0.300	3.00 (2.2)	0.339	0.89 (0.8)	0.335
Female	313	71.5	3.58 (2.8)		2.78 (2.2)		0.80 (0.8)	
Age (years)	438	81.5 (5.7)	0.05	0.312	0.04	0.377	0.05	0.281
IADL	438	35.2 (13.4)	0.20	<0.001	0.20	<0.001	0.15	0.002
ADL	438	1.5 (1.6)	0.00	0.933	−0.02	0.731	0.06	0.221
MMSE	438	16.2 (3.3)	−0.06	0.191	−0.06	0.176	−0.04	0.415
Behav. disturbances (no)	322	73.5	3.11 (2.6)	<0.001	2.39 (2.1)	<0.001	0.72 (0.8)	<0.001
Behav. disturbances (yes)	116	26.5	5.22 (2.6)		4.10 (2.1)		1.12 (0.8)	
*Caregivers' characteristics*								
Gender								
Male	148	33.8	3.20 (2.8)	0.011	2.52 (2.3)	0.030	0.68 (0.8)	0.005
Female	290	66.2	3.91 (2.7)		3.00 (2.2)		0.91 (0.8)	
Age (years)	438	61.4 (13.1)	0.08	0.089	0.08	0.114	0.07	0.121
Relationship								
Spouse	135	30.8	4.05 (2.8)	0.067	3.13 (2.2)	0.092	0.92 (0.8)	0.088
Child/child-in-law	244	55.7	3.39 (2.7)		2.64 (2.2)		0.75 (0.8)	
Other relative	59	13.5	3.95 (2.9)		3.02 (2.2)		0.93 (0.9)	
Education								
No title/basic level	132	30.1	4.05 (2.9)	0.063	3.12 (2.2)	0.096	0.93 (0.8)	0.094
Primary level	109	24.9	3.88 (2.9)		3.00 (2.2)		0.88 (0.8)	
Intermediate level	161	36.7	3.40 (2.8)		2.64 (2.2)		0.76 (0.8)	
High level	36	8.2	2.83 (2.2)		2.25 (1.9)		0.58 (0.7)	
MSPSS								
Total	438	62.1 (13.8)	−0.18	<0.001	−0.18	<0.001	−0.11	0.020
Family	438	23.4 (5.7)	−0.16	0.001	−0.16	0.001	−0.13	0.008
Friends	438	15.7 (7.6)	−0.16	0.001	−0.17	<0.001	−0.10	0.041
Others	438	23.1 (5.7)	−0.06	0.241	−0.07	0.166	−0.01	0.794
MCS-12	438	41.4 (11.8)	−0.38	<0.001	−0.34	<0.001	−0.38	<0.001
PCS-12	438	47.4 (9.7)	−0.17	<0.001	−0.16	0.001	−0.15	0.002
CBI	438	27.6 (16.8)	0.55	<0.001	0.51	<0.001	0.48	<0.001
HADS anxiety	438	6.8 (4.3)	0.38	<0.001	0.33	<0.001	0.40	<0.001
HADS depression	438	7.5 (4.4)	0.35	<0.001	0.31	<0.001	0.35	<0.001
CASE	438	3.7 (2.8)	—	—	—	—	—	—

Notes: values in column “%/mean” are expressed in either percentage or mean (±standard deviation, SD) where appropriate; values in column “CASE” are expressed in either *r* (Pearson's correlation coefficient) or mean (±standard deviation, SD) where appropriate; ADL = activities of daily living (normal score 6/6); IADL = instrumental activities of daily living (normal score 48/48); MMSE = Mini-Mental State Examination (normal score range 24–30); MSPSS = Multidimensional Scale of Perceived Social Support (range 4–28 for each subtotal score and 12–84 for the total score); MCS-12 = Mental Component Score (score range 0–100); PCS-12 = Physical Component Score (score range 0–100); CBI = Caregiver Burden Inventory (score range 0–96); HADS = Hospital and Anxiety Depression Scale (total score 21 each: 11–21 probable cases, 8–10 possible cases, and 0–7 no cases); CASE = Caregiver Abuse Screen; CASE total (score range 0–8); CASE interpersonal/physical-psychological abuse: items: 1, 2, 3, 4, 6, and 8 (score range 0–6); CASE neglect: items 5 and 7 (score range 0–2).

**Table 2:** Spearman's rank correlation coefficients with Bonferroni-adjusted significance level.

	Item 1	Item 2	Item 3	Item 4	Item 5	Item 6	Item 7	Item 8
Item 1	1							
Item 2	0.5230	1						
Item 3	0.6019	0.5173	1					
Item 4	0.4418	0.5446	0.4689	1				
Item 5	0.4745	0.4349	0.4555	0.3676	1			
Item 6	0.3713	0.369	0.4323	0.4537	0.4542	1		
Item 7	0.3577	0.3557	0.3776	0.3033	0.3647	0.4541	1	
Item 8	0.3932	0.4659	0.3758	0.6465	0.2893	0.4197	0.3364	1

Notes: all coefficients are statistically significant with *p* < 0.001.

**Table 3:** Factor analysis for CASE.

CASE items	% yes	Factor 1	Communality
Item 1: Do you sometimes have trouble making (name of person) control his/her temper or aggression?	36.3	0.7413	0.4505
Item 2: Do you often feel you are being forced to act out of character or do things you feel bad about?	46.6	0.7511	0.4359
Item 3: Do you find it difficult to manage (his/her) behavior?	44.1	0.7538	0.4318
Item 4: Do you sometimes feel that you are forced to be rough with (him/her)?	60.7	0.7539	0.4317
Item 5: Do you sometimes feel you cannot do what is really necessary or what should be done for (him/her)?	45.4	0.6719	0.5486
Item 6: Do you often feel you have to reject or ignore (him/her)?	41.8	0.6909	0.5227
Item 7: Do you often feel so tired and exhausted that you cannot meet (his/her) needs?	37.4	0.6069	0.6317
Item 8: Do you often feel you have to yell at (him/her)?	54.6	0.6937	0.5188

Notes: LR test: independent versus saturated: Chi-squared (28) = 1300.59 Prob > Chi-squared ≤ 0.0001.

**Table 4:** Multivariate linear regression model for the estimation of factors associated with higher CASE scores.

	CASE total			CASE interpers.			CASE neglect		
	*β*	S.E.	*p* value	*β*	S.E.	*p* value	*β*	S.E.	*p* value
*AD patients*									
Gender (female)	−0.11	0.25	0.665	−0.11	0.20	0.576	0.01	0.08	0.944
Age	0.01	0.02	0.658	0.00	0.02	0.790	0.00	0.01	0.461
IADL	−0.03	0.01	0.008	−0.02	0.01	0.026	−0.01	0.00	0.007
Behavioral disturbances (yes)	1.47	0.26	<0.001	1.22	0.21	<0.001	0.25	0.08	0.001
*Caregivers*									
Gender (female)	−0.06	0.25	0.813	−0.06	0.21	0.775	0.00	0.08	0.989
Age	0.01	0.01	0.560	0.00	0.01	0.747	0.00	0.00	0.295
MSPSS total	0.01	0.01	0.387	0.00	0.01	0.773	0.01	0.00	0.040
MCS-12	−0.02	0.01	0.127	−0.02	0.01	0.184	−0.01	0.00	0.146
PCS-12	0.00	0.01	0.924	0.00	0.01	0.978	0.00	0.00	0.703
CBI	0.08	0.01	<0.001	0.06	0.01	<0.001	0.02	0.00	<0.001
HADS anxiety	0.03	0.04	0.444	0.00	0.03	0.887	0.03	0.01	0.034
HADS depression	0.00	0.04	0.933	0.00	0.03	0.989	0.00	0.01	0.811
Constant	1.22	2.20	0.579	1.60	1.79	0.371	−0.38	0.68	0.574

Notes: S.E. = standard error.

## References

[1] World Health Organization (WHO) (2002). *The Toronto Declaration on the Global Prevention on Elder Abuse*.

[2] Dyer C. B., Connolly M. T., McFeeley P., Wallace R. B., Bonnie R. J. (2003). The clinical and medical forensics of elder abuse and neglect. *Elder Mistreatment: Abuse, Neglect, and Exploitation in an Aging America*.

[3] Lachs M. S., Pillemer K. A. (2015). Elder abuse. *The New England Journal of Medicine*.

[4] World Health Organization (WHO) (2011). *European Report on Preventing Elder Maltreatment*.

[5] Sethi D., Wood S., Mitis F. (2011). *European Report on Preventing Elder Maltreatment*.

[6] Kosberg J. I. (1988). Preventing elder abuse: identification of high risk factors prior to placement decisions. *The Gerontologist*.

[7] Jones J. S., Holstege C., Holstege H. (1997). Elder abuse and neglect: understanding the causes and potential risk factors. *The American Journal of Emergency Medicine*.

[8] Quinn M. J., Tomita S. K. (1997). *Elder Abuse and Neglect: Causes, Diagnosis, and Intervention Strategies*.

[9] Anthony E. K., Lehning A. J., Austin M. J., Peck M. D. (2009). Assessing elder mistreatment: instrument development and implications for adult protective services. *Journal of Gerontological Social Work*.

[10] Naughton C., Drennan J., Lyons I. (2012). Elder abuse and neglect in Ireland: results from a national prevalence survey. *Age and Ageing*.

[11] Melchiorre M. G., Chiatti C., Lamura G. (2013). Social support, socio-economic status, health and abuse among older people in seven European countries. *PLoS ONE*.

[12] Pillemer K., Burnes D., Riffin C., Lachs M. S. (2016). Elder abuse: global situation, risk factors, and prevention strategies. *The Gerontologist*.

[13] Acierno R., Hernandez M. A., Amstadter A. B. (2010). Prevalence and correlates of emotional, physical, sexual, and financial abuse and potential neglect in the United States: the National Elder Mistreatment Study. *American Journal of Public Health*.

[14] Burnes D., Pillemer K., Caccamise P. L. (2015). Prevalence of and risk factors for elder abuse and neglect in the community: a population-based study. *Journal of the American Geriatrics Society*.

[15] Gil A. P. M., Kislaya I., Santos A. J., Nunes B., Nicolau R., Fernandes A. A. (2015). Elder abuse in Portugal: findings from the first national prevalence study. *Journal of Elder Abuse and Neglect*.

[16] Lowenstein A., Eisikovits Z., Band-Winterstein T., Enosh G. (2009). Is elder abuse and neglect a social phenomenon? Data from the first national prevalence survey in Israel. *Journal of Elder Abuse and Neglect*.

[17] Biggs S., Manthorpe J., Tinker A., Doyle M., Erens B. (2009). Mistreatment of older people in the united kingdom: findings from the first national prevalence study. *Journal of Elder Abuse and Neglect*.

[18] Oh J., Kim H. S., Martins D., Kim H. (2006). A study of elder abuse in Korea. *International Journal of Nursing Studies*.

[19] Melchiorre M. G., Di Rosa M., Lamura G. (2016). Abuse of older men in seven European countries: a multilevel approach in the framework of an ecological model. *PLoS ONE*.

[20] Downes C., Fealy G., Phelan A., Donnelly N. A., Lafferty A. (2013). *Abuse of Older People with Dementia: A Review*.

[21] Pillemer K., Suitor J. J. (1992). Violence and violent feelings: what causes them among family caregivers?. *The Journals of Gerontology*.

[22] Marmolejo I. I. (2008). *Elder Abuse in the Family in Spain*.

[23] Comijs H. C., Pot A. M., Smit J. H., Bouter L. M., Jonker C. (1998). Elder abuse in the community: prevalence and consequences. *Journal of the American Geriatrics Society*.

[24] Cooney C., Howard R., Lawlor B. (2006). Abuse of vulnerable people with dementia by their carers: can we identify those most at risk?. *International Journal of Geriatric Psychiatry*.

[25] Cooper C., Selwood A., Blanchard M., Walker Z., Blizard G., Livingston G. (2009). Abuse of people with dementia by family carers: representative cross sectional survey. *British Medical Journal*.

[26] Vandeweerd C., Paveza G. J., Walsh M., Corvin J. (2013). Physical mistreatment in persons with Alzheimer's disease. *Journal of Aging Research*.

[27] Dong X., Chen R., Simon M. A. (2014). Elder abuse and dementia: a review of the research and health policy. *Health Affairs*.

[28] Boye F., Yan E. (2016). Abuse of older persons with dementia: a review of the literature. *Trauma, Violence, & Abuse*.

[29] Alzheimer Disease International The global voice on dementia. World Alzheimer Report 2015. The global impact of dementia. https://www.alz.co.uk/research/world-report-2015.

[30] Melchiorre M. G., Penhale B., Lamura G. (2014). Understanding elder abuse in Italy: perception and prevalence, types and risk factors from a review of the literature. *Educational Gerontology*.

[31] Lindert J., De Luna J., Torres-Gonzales F. (2013). Abuse and neglect of older persons in seven cities in seven countries in Europe—a cross-sectional community study. *International Journal of Public Health*.

[32] Sgritta G. B., Deriu F. *The Hidden Violence. Abuse and Mistreatment Against Older People*.

[33] Soares J. J. F., Barros H., Torres-Gonzales F. (2010). *Abuse and Health Among Elderly in Europe*.

[34] Melchiorre M. G., Di Rosa M., Lamura G. (2012). Il maltrattamento delle persone anziane in Italia: alcuni risultati dallo Studio Abuel (the mistratment of the elderly in Italy: some findings from the Abuel study). *Prospettive Sociali e Sanitarie*.

[35] Melchiorre M. G., Chiatti C., Lamura G. (2012). Tackling the phenomenon of elder abuse in Italy: a review of existing legislation and policies as a learning resource. *Educational Gerontology*.

[36] Molinelli A., Viale L., Landolfa M. C., De Stefano F. (2011). Old age as an “alternative” to illness: gerontological and medico-legal aspects. *Aging Clinical and Experimental Research*.

[37] Cooper C., Katona C., Finne-Soveri H., Topinková E., Carpenter G. I., Livingston G. (2006). Indicators of elder abuse: a cross-national comparison of psychiatric morbidity and other determinants in the Ad-HOC study. *The American Journal of Geriatric Psychiatry*.

[38] Ogioni L., Liperoti R., Landi F., Soldato M., Bernabei R., Onder G. (2007). Cross-sectional association between behavioral symptoms and potential elder abuse among subjects in home care in Italy: results from the silvernet study. *American Journal of Geriatric Psychiatry*.

[39] Mowlam A., Tennant R., Dixon J., McCreadie C. (2007). *UK Study of Abuse and Neglect of Older People: Qualitative Findings*.

[40] Fulmer T., Strauss S., Russell S. L. (2012). Screening for elder mistreatment in dental and medical clinics. *Gerodontology*.

[41] Cohen M. (2011). Screening tools for the identification of elder abuse. *Journal of Clinical Outcomes Management*.

[42] Cohen M. (2013). The process of validation of a three-dimensional model for the identification of abuse in older adults. *Archives of Gerontology and Geriatrics*.

[43] Worrilow W. (2015). *Physician Screening for Elder Abuse in the Emergency Department: A Literature Review*.

[44] Ferguson D., Beck C. (1983). H.A.L.F.—a tool to assess elder abuse within the family. *Geriatric Nursing*.

[45] Bass D. M., Anetzberger G. J., Ejaz F. K., Nagpaul K. (2001). Screening tools and referral protocol for stopping abuse against older Ohioans: a guide for service providers. *Journal of Elder Abuse and Neglect*.

[46] Fulmer T., Guadagno L., Dyer C. B., Connolly M. T. (2004). Progress in elder abuse screening and assessment instruments. *Journal of the American Geriatrics Society*.

[47] Phelan A., Treacy M. P. (2011). *A Review of Elder Abuse Screening Tools*.

[48] Reis M., Nahmiash D. (1998). Validation of the indicators of abuse (IOA) screen. *The Gerontologist*.

[49] Straus M. A. (1979). Measuring Intrafamily conflict and violence: the Conflict Tactics (CT) Scales. *Journal of Marriage and the Family*.

[50] Straus M. A., Hamby S. L., Boney-McCoy S., Sugarman D. B. (1996). The revised Conflict Tactics Scales (CTS2): development and preliminary psychometric data. *Journal of Family Issues*.

[51] Tronetti P. (2014). Evaluating abuse in the patient with Dementia. *Clinics in Geriatric Medicine*.

[52] Pérez-Rojo G., Nuevo R., Sancho M., Penhale B. (2015). Validity and reliability of the Spanish version of Caregiver Abuse Screen (CASE). *Research on Aging*.

[53] Reis M., Nahmiash D. (1995). Validation of the Caregiver Abuse Screen (CASE). *Canadian Journal on Aging*.

[54] Chiatti C., Masera F., Rimland J. M. (2013). The UP-TECH project, an intervention to support caregivers of Alzheimer's disease patients in Italy: study protocol for a randomized controlled trial. *Trials*.

[55] Barbabella F., Chiatti C., Rimland J. M. (2016). Socioeconomic predictors of the employment of migrant care workers by Italian families assisting older Alzheimer's disease patients: evidence from the Up-Tech study. *Journals of Gerontology, Series B: Psychological Sciences and Social Sciences*.

[56] Chiatti C., Furneri G., Rimland J. M. (2015). The economic impact of moderate stage Alzheimer's disease in Italy: evidence from the UP-TECH randomized trial. *International Psychogeriatrics*.

[57] Chiatti C., Rimland J. M., Bonfranceschi F. (2015). The UP-TECH project, an intervention to support caregivers of Alzheimer's disease patients in Italy: preliminary findings on recruitment and caregiving burden in the baseline population. *Aging and Mental Health*.

[58] Folstein M. F., Folstein S. E., McHugh P. R. (1975). ‘Mini-mental state’: a practical method for grading the cognitive state of patients for the clinician. *Journal of Psychiatric Research*.

[59] Chiatti C., Rimland J. M., Bonfranceschi F. (2015). The UP-TECH project, an intervention to support caregivers of Alzheimer's disease patients in Italy: Preliminary findings on recruitment and caregiving burden in the baseline population. *Aging and Mental Health*.

[60] American Association for Public Opinion Research (AAPOR) Standard Definitions. Final Dispositions of Case Codes and Outcome Rates for Surveys. http://www.aapor.org/AAPOR_Main/media/publications/Standard-Definitions2015_8theditionwithchanges_April2015_logo.pdf.

[61] Hirdes J. P., Curtin-Telegdi N., Poss J. W. (2010). *InterRAI Contact Assessment (CA) Form and User's Manual, 9.2.*.

[62] Morris J. N., Fries B. E., Morris S. A. (1999). Scaling ADLs within the MDS. *Journals of Gerontology—Series A Biological Sciences and Medical Sciences*.

[63] McDowell I. (2006). *Measuring Health: A Guide to Rating Scales and Questionnaires*.

[64] Gandek B., Ware J. E. (1998). Methods for validating and norming translations of health status questionnaires: the IQOLA Project approach. International Quality of Life Assessment. *Journal of Clinical Epidemiology*.

[65] Kodraliu G., Mosconi P., Groth N. (2001). Subjective health status assessment: Evaluation of the Italian version of the SF-12 health survey. Results from the MiOS project. *Journal of Epidemiology and Biostatistics*.

[66] Novak M., Guest C. (1989). Application of a multidimensional caregiver burden inventory. *The Gerontologist*.

[67] Marvardi M., Mattioli P., Spazzafumo L. (2005). The Caregiver Burden Inventory in evaluating the burden of caregivers of elderly demented patients: results from a multicenter study. *Aging Clinical and Experimental Research*.

[68] Zigmond A. S., Snaith R. P. (1983). The hospital anxiety and depression scale. *Acta Psychiatrica Scandinavica*.

[69] Zimet G. D., Dahlem N. W., Zimet S. G., Farley G. K. (1988). The multidimensional scale of perceived social support. *Journal of Personality Assessment*.

[70] Perel-Levin S. (2008). *Discussing Screening for Elder Abuse at Primary Health Care Level*.

[71] Reichenheim M. E., Paixão C. M., Moraes C. L. (2009). Reassessing the construct validity of a Brazilian version of the instrument Caregiver Abuse Screen (CASE) used to identify risk of domestic violence against the elderly. *Journal of Epidemiology and Community Health*.

[72] Schulz R., Belle S. H., Czaja S. J., McGinnis K. A., Stevens A., Zhang S. (2004). Long-term care placement of dementia patients and caregiver health and well-being. *Journal of the American Medical Association*.

[73] VandeWeerd C., Paveza G. J. (2006). Verbal mistreatment in older adults: a look at persons with Alzheimer's disease and their caregivers in the State of Florida. *Journal of Elder Abuse and Neglect*.

[74] Pérez-Rojo G., Izal M., Montorio I., Penhale B. (2009). Risk factors of elder abuse in a community dwelling Spanish sample. *Archives of Gerontology and Geriatrics*.

[75] Wiglesworth A., Mosqueda L., Mulnard R., Liao S., Gibbs L., Fitzgerald W. (2010). Screening for abuse and neglect of people with dementia. *Journal of the American Geriatrics Society*.

[76] Fauth E. B., Gibbons A. (2014). Which behavioral and psychological symptoms of dementia are the most problematic? Variability by prevalence, intensity, distress ratings, and associations with caregiver depressive symptoms. *International Journal of Geriatric Psychiatry*.

[77] Kosberg J. I., Nahmiash D., Baumhoer L. A., Bell S. C. (1996). Characteristics of victims and perpetrators milieus of abuse and neglect. *Abuse, Neglect and Exploitation of Older Persons: Strategies for Assessment and Intervention*.

[78] Wolf R. S., Pillemer K. A. (1989). *Helping Elderly Victims: The Reality of Elder Abuse*.

[79] Schiamberg L. B., Gans D. (2000). Elder abuse by adult children: an applied, ecological framework for understanding contextual risk factors and the intergenerational character of quality of life. *International Journal of Aging and Human Development*.

[80] Kilburn J. C. (1996). Network effects in caregiver to care-recipient violence. *Journal of Elder Abuse and Neglect*.

[81] Schulz R., Visintainer P., Williamson G. M. (1990). Psychiatric and physical morbidity effects of caregiving. *Journals of Gerontology*.

[82] Baumgarten M., Battista R. N., Infante-Rivard C., Hanley J. A., Becker R., Gauthier S. (1992). The psychological and physical health of family members caring for an elderly person with dementia. *Journal of Clinical Epidemiology*.

[83] Miller L. S., Lewis M. S., Williamson G. M. (2006). Caregiver cognitive status and potentially harmful caregiver behavior. *Aging and Mental Health*.

[84] Johannesen M., Logiudice D. (2013). Elder abuse: a systematic review of risk factors in community-dwelling elders. *Age and Ageing*.

[85] Henderson D., Buchanan J. A., Fisher J. E., Schewe P. A. (2002). Violence and the elderly population: issues for prevention. *Preventing Violence in Relationships: Interventions Across the Life Span*.

[86] Di Rosa M., Greco C., Melchiorre M. G., Senin U., Bartorelli L., Salvioli G. The home care. *The Older Old: Taking Care*.

[87] Principi A., Lamura G., Sirolla C. (2014). Work restrictions experienced by midlife family care-givers of older people: evidence from six European countries. *Ageing and Society*.

[88] Coyne A. C., Reichman W. E., Berbig L. J. (1993). The relationship between dementia and elder abuse. *American Journal of Psychiatry*.

[89] Hansberry M. R., Chen E., Gorbien M. J. (2005). Dementia and elder abuse. *Clinics in Geriatric Medicine*.

[90] Drossel C., Fisher J. E., Mercer V. (2011). A DBT skills training group for family caregivers of persons with dementia. *Behavior Therapy*.

[91] Naik A. D., Kunik M. E., Cassidy K. R., Nair J., Coverdale J. (2010). Assessing safe and independent living in vulnerable older adults: perspectives of professionals who conduct home assessments. *Journal of the American Board of Family Medicine*.

[92] Lachs M. S., Pillemer K. A. (1995). Current concepts-abuse and neglect of elderly persons. *New England Journal of Medicine*.

[93] Colerick E. J., George L. K. (1986). Predictors of institutionalization among caregivers of patients with Alzheimer's disease. *Journal of the American Geriatrics Society*.

[94] Homer A. C., Gilleard C. (1990). Abuse of elderly people by their carers. *British Medical Journal*.

[95] Moak G. S. (1990). Characteristics of demented and non-demented geriatric admissions to a state hospital. *Hospital & Community Psychiatry*.

[96] Chenoweth B., Spencer B. (1986). Dementia: the experience of family caregivers. *The Gerontologist*.

[97] Desai A. K., Grossberg G. T. (2001). Recognition and management of behavioral disturbances in dementia. *Primary Care Companion to the Journal of Clinical Psychiatry*.

[98] Shah J., Wadoo O., Latoo J. (2010). Psychological distress in carers of people with mental disorders. *British Journal of Medical Practitioners (BJMP)*.

[99] Lochhead J. D., Nelson M. A., Maguire G. A. (2016). The treatment of behavioral disturbances and psychosis associated with dementia. *Psychiatria Polska*.

[100] Lachs M. S., Williams C., O'Brien S., Hurst L., Horwitz R. (1997). Risk factors for reported elder abuse and neglect: a nine-year observational cohort study. *The Gerontologist*.

[101] Yan E. (2014). Abuse of older persons with dementia by family caregivers: results of a 6-month prospective study in Hong Kong. *International Journal of Geriatric Psychiatry*.

[102] O'Loughlin A., Duggan J. (1998). Abuse, neglect and mistreatment of older people: an exploratory study.

[103] Montorio Cerrato I., Izal Fernández de Trocóniz M., López López A., Sánchez Colodrón M. (1998). The Burden Interview. Usefulness and validity of the burden concept. *Anales de Psicologia*.

[104] Pot A. M., Van Dyck R., Jonker C., Deeg D. J. H. (1996). Verbal and physical aggression against demented elderly by informal caregivers in the Netherlands. *Social Psychiatry and Psychiatric Epidemiology*.

[105] Lee M., Kolomer S. (2005). Caregiver burden, dementia, and elder abuse in South Korea. *Journal of Elder Abuse and Neglect*.

[106] Cooper C., Manela M., Katona C., Livingston G. (2008). Screening for elder abuse in dementia in the LASER-AD study: prevalence, correlates and validation of instruments. *International Journal of Geriatric Psychiatry*.

[107] Brodaty H., Donkin M. (2009). Family caregivers of people with dementia. *Dialogues in Clinical Neuroscience*.

[108] Ory M. G., Hoffman R. R., Yee J. L., Tennstedt S., Schulz R. (1999). Prevalence and impact of caregiving: a detailed comparison between dementia and nondementia caregivers. *The Gerontologist*.

[109] Mohide E. A., Torrance G. W., Streiner D. L., Pringle D. M., Gilbert R. (1988). Measuring the wellbeing of family caregivers using the time trade-off technique. *Journal of Clinical Epidemiology*.

[110] Dong X., Simon M. A. (2010). Gender variations in the levels of social support and risk of elder mistreatment in a Chinese community population. *Journal of Applied Gerontology*.

[111] Reay A. M. C., Browne K. D. (2001). Risk factor characteristics in carers who physically abuse or neglect their elderly dependants. *Aging and Mental Health*.

[112] Cooper C., Katona C., Orrell M., Livingston G. (2006). Coping strategies and anxiety in caregivers of people with Alzheimer's disease: the LASER-AD study. *Journal of Affective Disorders*.

[113] Cooper C., Balamurali T. B. S., Livingston G. (2007). A systematic review of the prevalence and covariates of anxiety in caregivers of people with dementia. *International Psychogeriatrics*.

[114] Redinbaugh E. M., Baum A., DeMoss C., Fello M., Arnold R. (2002). Factors associated with the accuracy of family caregiver estimates of patient pain. *Journal of Pain and Symptom Management*.

[115] Mittelman M. S., Roth D. L., Haley W. E., Zarit S. H. (2004). Effects of a caregiver intervention on negative caregiver appraisals of behavior problems in patients with Alzheimer's Disease: results of a randomized trial. *Journals of Gerontology—Series B Psychological Sciences and Social Sciences*.

[116] Mittelman M. S., Roth D. L., Coon D. W., Haley W. E. (2004). Sustained benefit of supportive intervention for depressive symptoms in caregivers of patients with alzheimer's disease. *American Journal of Psychiatry*.

[117] Lee M. (2008). Caregiver stress and elder abuse among Korean family caregivers of older adults with disabilities. *Journal of Family Violence*.

[118] Sev'er A. (2009). More than wife abuse that has gone old: a conceptual model for violence against the aged in Canada and the US. *Journal of Comparative Family Studies*.

[119] de Donder L., Luoma M.-L., Penhale B. (2011). European map of prevalence rates of elder abuse and its impact for future research. *European Journal of Ageing*.

[120] De Donder L., De Witte N., Brosens D., Dierckx E., Verté D. (2015). Learning to detect and prevent elder abuse: the need for a valid risk assessment instrument. *Procedia - Social and Behavioral Sciences*.

[121] de Oliveira A. C., Rocha I. P., do Nascimento Mota F. R., Falcão Júnior J. S. P., da Silva M. J., Victor J. F. (2014). Screening of potentially violent attitudes against elderly committed by their caregivers. *Journal of Nursing UFPE On Line*.

[122] Selwood A., Cooper C., Owens C., Blanchard M., Livingston G. (2009). What would help me stop abusing? the family carer's perspective. *International Psychogeriatrics*.

[123] Dong X. Q., Chang E.-S., Wong E., Wong B., Simon M. A. (2011). How do U.S. Chinese older adults view elder mistreatment? Findings from a community-based participatory research study. *Journal of Aging and Health*.

[124] Peri K., Fanslow J., Hand J., Parsons J. (2009). Keeping older people safe by preventing elder abuse and neglect. *Social Policy Journal of New Zealand*.

[125] Ayalon L. (2011). Abuse is in the eyes of the beholder: using multiple perspectives to evaluate elder mistreatment under round-the-clock foreign home carers in Israel. *Ageing and Society*.

[126] Baker P. R. A., Francis D. P., Hairi N. N., Othman S., Choo W. Y. (2016). Interventions for preventing abuse in the elderly. *Cochrane Database of Systematic Reviews*.

[127] Dong X. (2014). Elder abuse: research, practice, and health policy. The 2012 GSA Maxwell pollack award lecture. *The Gerontologist*.

